# Psychopathic traits mediate guilt-related anterior midcingulate activity under authority pressure

**DOI:** 10.1038/s41598-021-94372-5

**Published:** 2021-07-21

**Authors:** Yawei Cheng, Judith Chou, Róger Marcelo Martínez, Yang-Teng Fan, Chenyi Chen

**Affiliations:** 1 Department of Physical Medicine and Rehabilitation, National Yang Ming Chiao Tung University Hospital, Yilan, Taiwan; 2 Institute of Neuroscience and Brain Research Center, National Yang Ming Chiao Tung University, Taipei, Taiwan; 3 Department of Education and Research, Taipei City Hospital, Taipei, Taiwan; 4 Graduate Institute of Injury Prevention and Control, College of Public Health, Taipei Medical University, Taipei, Taiwan; 5 School of Psychological Sciences, National Autonomous University of Honduras, Tegucigalpa, Honduras; 6 Graduate Institute of Medicine, Yuan Ze University, Taoyuan City, Taiwan; 7 Research Center of Brain and Consciousness, Shuang-Ho Hospital, Taipei Medical University, New Taipei City, Taiwan; 8 Graduate Institute of Mind, Brain and Consciousness, College of Humanities and Social Sciences, Taipei Medical University, Taipei, Taiwan; 9 Psychiatric Research Center, Wan Fang Hospital, Taipei Medical University, Taipei, Taiwan

**Keywords:** Neuroscience, Cognitive neuroscience, Emotion, Motivation, Social behaviour, Social neuroscience

## Abstract

Coercive power has different effects on individuals, and which were unable to be fully addressed in Milgram’s famous studies on obedience to authority. While some individuals exhibited high levels of guilt-related anxiety and refused orders to harm, others followed coercive orders throughout the whole event. The lack of guilt is a well-known characteristic of psychopathy, and recent evidence portrays psychopathic personalities on a continuum of clustered traits, while being pervasive in a significant proportion in the population. To investigate whether psychopathic traits better explain discrepancies in antisocial behavior under coercion, we applied a virtual obedience paradigm, in which an experimenter ordered subjects to press a handheld button to initiate successive actions that carry different moral consequences, during fMRI scanning. Psychopathic traits modulated the association between harming actions and guilt feelings on both behavioral and brain levels. This study sheds light on the individual variability in response to coercive power.

## Introduction

In 1963, Milgram published the findings of his now famous experiments on obedience to authority^1^. Compliance to cause other people harm was attributed to the psychological phenomenon of “diffusion of responsibility”. Yet under the same social context, not all participants were coerced into delivering harm, which suggests other factors at play. While 35% of the participants successfully disobeyed orders to finish the “experiment”, in which participants believed they were administering harmful electrical impulses to others, the rest completed the task, with their compliance being attributed to the expert status and assumed power displayed by the experimenter^2^. In real-life situations, individuals who obey coercive orders to harm also exhibit differences in emotional responses to their immoral actions^2^. Using historical examples, during the World War II Nuremberg trials, a number of war criminals took their own lives out of guilt-like anxiety before the trials even began, whereas others attended the whole prosecution with seeming indifference^3^. One famous example of the latter, and although his trial took place several years later in Jerusalem, was that of Adolf Eichmann. His lack of remorse and guilt was cemented on the excuse that he was “just obeying orders”, with his motivations being solely those of climbing up the economic, social, and political ladder. All shallow goals considering the horrors he had to commit in order to reach them, and by which Hannah Arendt coined the now famous phrase “the banality of evil”^4^. But then, these observed differences spark the question of whether this wide spectrum of individual differences can predict the outcomes of decision-making under coercion better than social context, or in other words, how individuals decide whether to obey an order issued by an authoritative figure that causes direct harm to others. Although coercion-altered Event-Related-Potentials (ERPs) have been found to be associated with the auditory N1, induced by an implicit intentional binding paradigm, the neural mechanisms underlying the link between coercive violence, psychopathic traits, and guilt remain elusive^5^.

Guilt has long been identified as a fundamental moral emotion, with a clear influence in driving moral behavior; individuals tend to avoid wrongdoings against others to circumvent unpleasant feelings of guilt^6^. The current literature offers two types of guilt—altruistic and deontological; the first derives from harming or wronging others, and the latter results from violating self-determined moral values^7^. Guilt levels can predict prosocial behavior in adolescents^8^. This effect maintains even within incarcerated populations, as guilt levels were negatively correlated with recidivism^9^. Consequently, researchers have explained antisocial behavior with contextual mechanisms that lessen feelings of altruistic guilt, such as the diffusion of responsibility in coercive situations^1,10,11^.

However, the presence of guilt does not consistently produce prosocial consequences. In contexts wherein no clear dyadic relationship exists, i.e. lacking two actors such as a transgressor and a victim, guilt led to immoral decisions^12^. More specifically, guilt propelled people to offer money to help a disadvantaged person even at the expense of hurting others who were also in need of the money. The findings delineate that the relationship between guilt and morality is not unidirectional, and that guilt can lead to unjust outcomes. On the other hand, the lack of guilt, as observed in individuals with psychopathy, does not consistently result in a life of criminality and immorality^13^. Researchers argue that psychopathic individuals are well integrated in society, and some studies even demonstrated that individuals with psychopathic traits performed better—with more moral choices—than controls on moral dilemma tasks^14,15^. As the presence of guilt cannot deter all antisocial behavior, and the lack of guilt can still produce moral actions, it is very likely that the relationships between guilt, psychopathic traits, and antisocial behavior is highly complex and worth exploring.

Psychopathy is associated with a cluster of traits including manipulativeness, dishonesty, narcissism, superficial charm, reckless risk-taking, impulsive antisocial behavior and, arguably one of the most characteristic traits, the lack of guilt^16,17^. Behavioral studies have established a negative correlation between guilt-related skills and psychopathic traits; for example, psychopathic individuals exhibited difficulty in attributing guilt to the correct facial expression^18,19^. However, there still exists a deficit in the exploration in regards to the neural correlates of guilt and psychopathy. Previous studies on healthy individuals have found neural correlates of hypothetical and recollected guilt^7,20–25^, whereas one study prompted present-time moral emotions^26^.

As there is a great need for non-symptom-based methods to detect psychopathy to optimize predictive validity, using symptoms or criminal behavior to diagnose psychopathy is not conducive for preventing antisocial behavior^27^. After first transgressions, individuals are at a higher risk of committing subsequent offences^28^, which further highlights the value of early detection. Furthermore, the updated perspective on psychopathy, which is aligned with the dominant perspective on personality disorders more broadly, is that individuals with psychopathy may be better conceived as a continuum in the population, rather than a discrete category or unique taxon (i.e., a distinct subtype of individuals) see^29^. Given such, there is a possibility that current measures can only detect psychopathy that exceeds a certain threshold and overlooks others with milder forms. There is a possibility that everyday antisocial behavior can be explained by individual psychopathy. Consequently, exploring the relationship between the neural correlates of guilt and psychopathy is important.

Here, the study explores whether psychopathic traits within healthy subjects can predict harming behavior under coercion. We employed an fMRI virtual paradigm inspired on Milgram’s experiments in order to elicit first-hand guilt experience in relation to psychopathic traits. We predict that, under coercion, participants scoring higher on psychopathy measures will be more willing and hence quicker to allow for harming actions to occur, alongside lower experienced guilt. Furthermore, we explore the linkage between guilt and psychopathy at large, as well as identify any neural pathways responsible for their relationship. Harming trials will result in significant activations of guilt-related neural regions. We evaluate whether everyday psychopathic behavior in sub-clinical participants can be captured in the laboratory, as well as examine its guilt-related brain mediators.

## Materials and methods

### Participants

To estimate the sample size needed for examining the behavioral and neurophysiological correlates of psychopathic traits among non-clinical population, we conducted G*power 3.1^30^ based on the data from a previous study^31^. The calculated effect size *r* for the primary outcomes ranged from 0.40 to 0.62, corresponding to an average effect size *ρ* of 0.3. To have 80% power to detect a true difference, 64 participants are required with a 2-sided type I error of 0.05. Sixty-one neurotypical volunteers (32 females) between 20 and 30 years of age (23 ± 3.3 years) were recruited through an online survey disseminated through social media. Because of potential medical complications and highly atypical scores on the PPI-R validity subscale, five participants were excluded, so that only the data collected from 56 out of 61 participants were processed (Table 1). All participants had normal or corrected-normal visual acuity. None of them had any history of neurological or psychiatric disorders, and all were free of medication at the time of testing. Each participant was briefed and each signed an informed consent form at the start of the experiment. The study was approved by the institutional review board of National Yang-Ming University and conducted in accordance with the Declaration of Helsinki.

**Table 1:** Demographic variables of the participants in the study.

	High PPI-R subgroup	Low PPI-R subgroup		The total group
	(*N* = 29)	(*N* = 27)	*P* value	
**Sex**				
Male	29 (52%)	27 (48%)	.553	56 (50%)
Age	23.24 (2.52)	25.56 (2.33)	.3	22.91 (2.43)
Harming RTs (raw)	1212.99 (484.8)	1251.5 (755.37)	.168	1231.55 (624.31)
Neutral RTs (raw)	1121.9 (406.49)	1167.20 (613.54)	.184	1143.74 (512.44)
Harming RTs (LOG10-transformed)	3.05 (0.17)	3.03 (0.24)	.214	3.04 (0.2)
Neutral RTs (LOG10-transformed)	3.02 (0.15)	3.02 (0.2)	.234	3.02 (0.18)
Cold heartedness (C)	31.88 (4.68)	31.07 (4.28)	.850	31.49 (4.47)
Machiavellian Egocentricity (ME)	47.83 (6.89)	43.56 (4.78)	.036	45.77 (6.3)
Rebellious Nonconformity (RN)	41.34 (6.09)	34.81 (6.22)	.905	38.2 (6.93)
Blame Externalization (BE)	33.21 (6.17)	30.93 (4.09)	.180	32.11 (5.35)
Carefree Nonplanfulness (CN)	37.24 (6.25)	34.07 (7.79)	.322	35.71 (7.15)
Social Influence (SOI)	46.86 (7.21)	40.33 (7.49)	.814	43.71 (7.99)
Fearlessness (F)	38.66 (5.91)	31.93 (5.36)	.644	35.41 (6.55)
Stress Immunity (STI)	31.66 (8.13)	28.15 (4.55)	.002	29.96 (6.82)
Total PPI score	307.83 (12.29)	275.19 (14.39)	< .001	292.09 (21.11)

Participants were divided into subgroups of relatively High (*n* = 29) and Low (*n* = 27) PPI-R total scores based on mean and median split. PPI-R, Psychopathic Personality Inventory-Revised.

Data are presented as mean (SD) or number of participants (%).

### Procedures

Participants filled in the Psychopathic Personality Inventory-Revised (PPI-R), and underwent fMRI scanning. The PPI-R is a self-reported questionnaire designed by Lilienfeld and Widows^32^ predominantly used on non-clinically-diagnosed individuals to measure psychopathic traits. The 8 subscales include: Machiavellian Egocentricity (ME); Rebellious Non-conformity (RN); Blame Externalization (BE); Carefree Non-planfulness (CN); Social Influence (SOI); Fearlessness (F); Stress Immunity (STI); Cold heartedness (C).

**Figure 1:**
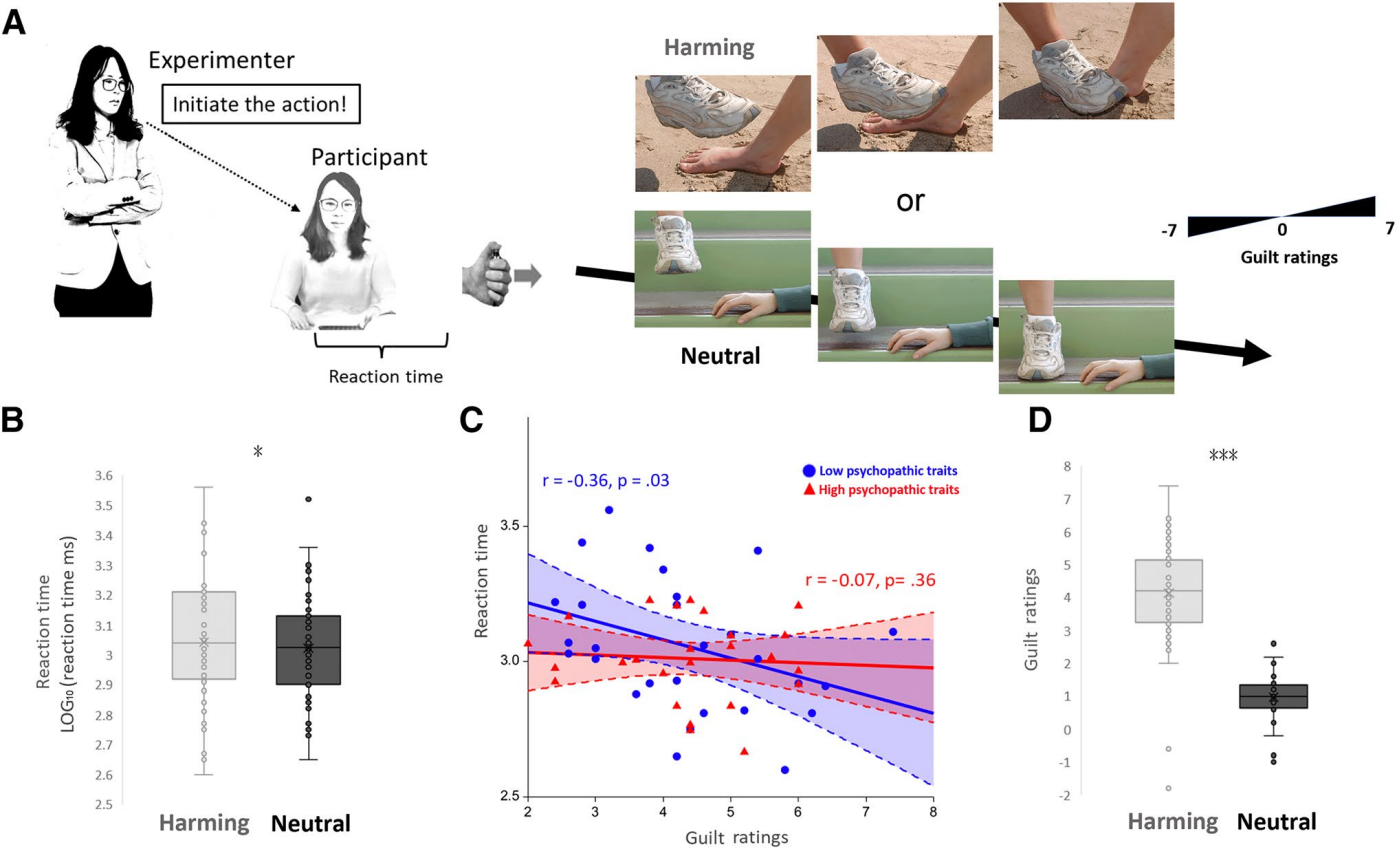
Experimental setup and scenario effect on the reaction time and guilt ratings to coercive commands. (**A**) Schematic representation of the paradigm for coercive commands. The experimenter ordered the participant to commit harming or neutral behavior by pressing a trigger button in a virtual computerized program along with visual feedback of moral scenarios. (**B**). The reaction time (RT) in harming was longer than that in neutral (*P* = .04). Participants showed less obedience (i.e., longer RTs) to initiate harming (3.042 ± 0.027, mean ± SE) than to initiate neutral (3.022 ± 0.024) actions. (**C**) While there was an overall significant correlation between reaction time and guilt ratings found in the whole group analysis, indicating that participants who obeyed harming orders more promptly (i.e., shorter RTs) were reported with stronger feelings of guilt, this effect was mainly driven from participants who scored lower on psychopathic fearlessness traits (Low vs. High psychopathy: *r* =  − 0.36, *P* = .03 vs, *r* =  − 0.07, *P* = .36). (**D**) Under coercion, higher guilt ratings were reported for harming (4.132 ± 0.212), as compared to neutral actions (0.971 ± 0.086).

To elicit and measure experienced guilt, we designed a virtual obedience paradigm inspired by prior studies on obedience to authority^5,33–36^, in which an experimenter ordered a subject to inflict harm to a third party (Fig. 1A). During fMRI scanning, participants watching the first image of a morally-laden scenario mini clip were forced (ordered via textual instructions) to press a button in order to initiate the successive actions that carry different moral consequences, including harming and neutral actions, along with visual feedback of such moral scenarios. This task was based on the stimuli used in previous research wherein each moral action was animated by three still images shown consecutively with no duration limit set for the 1st image, but a 200 ms duration set for the 2nd image, and a 1000 ms duration set for the 3rd image^37,38^. We employed animations in two conditions (1) a person who is taking an action to physically harm another person (harming), and (2) baseline stimuli depicting a person carrying out an action that is irrelevant to another one (neutral). The faces of the protagonists were not visible to ensure that no emotional reactions could be seen by the participants. The participant would observe the first image (with no duration limit set, as to gauge for reaction time) of the animation, then would have to press the button following the coercive order to induce the remaining two images to play out. While the block design was implemented with ON blocks during the action conditions and OFF blocks sandwiched in-between, we gave textual instructions to participants right before the beginning of each ON block and forced them to initiate harming or neutral actions. Stimuli were presented with the E-prime software (Psychology Software Tools, Inc., Pittsburgh, PA) and an MRI compatible goggle (VisualStim Controller, Resonance Technology Inc.)

Outside the MRI scanner, participants completed a random sample of the virtual obedience paradigm (5 trials of each moral action condition) and were asked to indicate how much guilt the actions made them feel. The ratings were on a − 7 to 7 Likert scale from “rewarding (− 7)”, passing through “neutral (0)”, to “very much guilty (7)”.

### Validity of the virtual obedience paradigm

We conducted a complementary behavioral study to compare the feelings of coercion and their respective RTs in “coercive” and “free-will” groups. In this additional behavioral study, which counted with an independent group of newly collected participants (n = 50), half of the participants (n = 25) was randomly assigned to the “coercion” group, and half of them was randomly assigned to the “free-will” group. In the “free-will” group, participants were first informed about the instruction as follows “you can decide to be the agent to initiate the following harming or neutral actions by pressing the button or to be just an observer to watch the actions to be played out”. Each participant could freely choose to be an agent or an observer by pressing the button. If a participant chose to be an observer in the following trials, the animation that was comprised of three images would be played out with a duration of 3000, 200, and 1000 ms, respectively, without measuring the RTs. In the “coercion” group, the same textual instruction (as in the virtual obedience paradigm) “initiating the harming/or neutral actions” was applied. At the end of each trial, participants were asked to indicate how much the action would violate their own will. The ratings were on a 1 to 7 Likert scale, from “not feeling at all violated” to “feeling very violated”.

### Functional MRI scanning

To support the virtual obedience paradigm, an fMRI block design was implemented with ON blocks during the action conditions (harming and neutral blocks) and OFF blocks sandwiched in-between. Each run consisted of 4 active ON-blocks (2 neutral and 2 harming) in a pseudo-randomized sequence and had two runs. Right before the beginning of each ON block, we gave textual instructions to participants and forced them to initiate harming or neutral actions. The block condition was specified to prime participants’ guilt feelings.

During each ON block of 3 trials (harming trial: 2431.6 ± 624.3 ms; neutral trial: 2343.7 ± 512.4 ms), inter-trial intervals of 2200-ms were delivered with singular white fixation crosses centered on the screen. The ON block duration was defined from the moment participants pressed the button for the first trial within the block and up until the showing of the last image of the third and final trial. The total length of functional EPI ranged from 122 to 158 scans with the mean and standard deviation of 134.04 and 7.37.

Participants entered a 3 T Siemens MRI scanner (Magnetom Tim Trio, Erlangen, Germany) equipped with a high-resolution 12-channel head array coil. All changes in blood oxygenation level-dependent (BOLD) T2* weighted MR signals were measured by a gradient echo-planar imaging (EPI) sequence (repetition time TR = 2200 ms, echo time TE = 30 ms, FOV = 220 × 220 mm^2^, flip angle = 90°, matrix size = 64 × 64, 36 transversal slices, voxel size = 3.4 × 3.4 × 3.0 mm^3^, no gaps). EPI volume images were acquired along the AC–PC plane, and high-resolution structural MR images were acquired with a 3D magnetization-prepared rapid gradient echo sequence (3D-MPRAGE; TR = 2530 ms, TE = 3.5 ms, FOV = 256 × 256 mm^2^, flip angle = 7°, TI = 1100 ms, matrix size = 256 × 256, 192 sagittal slices, voxel size = 1.0 × 1.0 × 1.0 mm^3^, no gaps); and as used in another study^39^.

### Imaging data analysis

All functional and structural images were preprocessed in MATLAB 9.0 (The MathWorks, Inc., Natick, Massachusetts) with Statistical Parametric Mapping (SPM) 12 (Wellcome Department of Imaging Neuroscience, London). First, all EPI images were manually reoriented to the respective T1 images of each subject for alignment purposes before slice timing and realignment. Then, the images were coregistered to the respective T1 and mean EPI image files to further prevent misalignment. After the individual brain activation templates were created, EPI images were normalized into Montreal Neurological Institute (MNI) stereotaxic space and smoothed at 8 mm full-width at half-maximum (FWHM) Gaussian kernel. A high-pass frequency filter (128-s cutoff) was applied to the time series.

A first-level analysis was conducted using a general linear model to isolate the conditions of interest, Harming (13.9 ± 0.6 s) and Neutral blocks (13.6 ± 0.5 s), were modeled separately with the duration of the participant’s reaction time beginning at the onset of each ON block. The null event (fixation) was modeled with the duration of 13.2 ± 4.4 s. Movement parameters from the realignment output were included as regressors of no interest. The two regressors modelling the Harming and Neutral conditions were convolved with the hemodynamic response function. Parameter estimates of these two conditions were contrasted in order to yield one contrast image per participant for the Harming vs. Neutral contrast. These contrasts were used for the second-level regression analysis to explore activations that correlate with psychopathy and guilt. Whole-brain activations were reported at a family-wise error- (FWE-) corrected *P* < 0.05 level (unless otherwise noted) with a cluster level minimum of five voxels.

To explore the extent to which neural responses involved in harming others were modulated by psychopathic traits and subjective feeling, we conducted the whole-brain multiple regression analyses with the PPI-R scores and guilt ratings as a continuous variable, respectively (FWE rate at *P* < 0.05).

Regions of interest (ROIs) activations were extracted using the MarsBaR toolbox (http://marsbar.sourceforge.net/) installed in SPM12. The ROI for the anterior midcingulate cortex (aMCC: − 16, 6, 38) was reported for significant contrast image peaks within 10 mm of the priori coordinates that were determined on the basis of previous findings^7,20,22,24–26,40^ of guilt, as well as in one recent fMRI study which employed an obedience under coercion paradigm^34^.

### Mediation analyses

Mediation Effect Parametric Mapping was used to test specific hypotheses about brain-behavior relationships^41–43^. Here, intrigued by previous literature, while very different behavioral patterns and emotional consequences could be observed in agents under coercion [~ 60% of total participants who might suffer from various degree of guilt/and anxiety, from extreme anxious to not at all (e.g. the notorious case of Eichmann) were prepared to inflict fatal voltages to victims]^1^, we were curious whether the extent of experienced guilt that was induced during coercive harming could be modulated by individual differences of psychopathy, and hence psychopathic traits modulated/or mediated the relationship between guilt and brain ROI activity.

In the mediation analysis model, path a coded the link in which the predictor variable must be related to the mediator. The mediator was psychopathic traits (total PPI-R). Path b coded the link in which the mediator must be directly related to the outcome. The mediation effect (a ∗ b) must be significant, which amounts to a statistical test on the product of the a and b path coefficients. Equivalently, the test for the predictor-outcome relationship would be significantly reduced by the inclusion of the mediator in the path model. We refer the overall predictor outcome relationship as the c effect, and control the direct effect for the mediator as c0. The a ∗ b effect was to test the significance of c–c′; and as stated in another study^44^.

## Results

### Reaction times and guilt ratings

The reaction time (RT) to initiating behaviors under coercion was LOG-transformed and subject to a one-factorial repeated measures ANOVA with two levels (scenario: harming vs. neutral) (Fig. 1B). There was a main effect of scenario (*F*_1, 55_ = 4.43, *P* = 0.04, η^2^ = 0.075), indicating that participants were taking longer time to initiate harming (3.042 ± 0.027, mean ± SE) than neutral (3.022 ± 0.024) actions. The subjective guilt ratings were also subject to a one-factorial repeated measures ANOVA with two levels (scenario: harming vs. neutral). The main effect of scenario (*F*_1, 55_ = 138.73, *P* < 0.001, η^2^ = 0.716) revealed higher guilt feeling to harming (4.132 ± 0.212), as compared to neutral behaviors (0.971 ± 0.086), under coercion (Fig. 1D). The RTs of harming were significantly correlated with the guilt ratings (*r* =  − 0.28, *P* = 0.036).

### Reaction times, guilt ratings, and psychopathic traits

To examine the relationship between RTs, guilt feelings, and psychopathic traits, we conducted multiple regression analyses (Table 2). A first model including harming RTs as the dependent variable (*F*_9, 46_ = 2.27, *P* = 0.034) showed that the Rebellious Nonconformity (β = 0.377, *p* = 0.011) and Fearlessness (β =  − 0.327, *p* = 0.028) subscales predicted RTs to follow harming orders, explaining 31% of the variance. A model for neutral RTs (*F*_9, 46_ = 1.815, *P* = 0.091) also showed significant correlations with the Rebellious Nonconformity (β = 0.386, *p* = 0.012) and Fearlessness (β =  − 0.316, *p* = 0.039) subscales. Higher Rebellious Nonconformity scores were associated with longer RTs, whereas higher Fearlessness scores were associated with shorter RTs to commit harming. We carried out a third model with guilt ratings as the dependent variable. While guilt ratings were negatively correlated with total PPI-R scores (*r* =  − 0. 29, *P* = 0.028), indicating less guilt in individuals with higher psychopathic traits, each subscale did not independently predict guilt ratings (*F*_9, 46_ = 1.38, *P* = 0.224).

**Table 2:** Standardized coefficients of the multiple regression models.

Predictors	Model I DV:		Model II DV:		Model III DV:	
	Harming RTs		Guilt ratings		Neutral RTs	
	β	*p*	β	*p*	β	*p*
Gender	0.231	.096	− 0.248	.094	0.189	.185
Cold heartedness (C)	− 0.22	.116	− 0.023	.876	− 0.228	.115
Machiavellian Egocentricity (ME)	0.179	.252	− 0.209	.211	0.104	.52
Rebellious Nonconformity (RN)	0.377	**.011**	− 0.22	.154	0.386	**.012**
Blame Externalization (BE)	− 0.092	.574	− 0.026	.883	− 0.049	.769
Carefree Nonplanfulness (CN)	− 0.105	.466	− 0.017	.91	− 0.045	.759
Social Influence (SOI)	− 0.283	.091	− 0.035	.844	− 0.191	.265
Fearlessness (F)	− .327	**.028**	.144	.354	− 0.316	**.039**
Stress Immunity (STI)	.201	.239	− .134	.459	.161	.359

While we found an overall trend between shorter RTs and stronger guilt ratings, this association might vary as a function of psychopathic traits. Based on a priori knowledge that suggested a widely different nature between high and low psychopathy^45^, we divided subgroups of relatively High (*n* = 29) and Low (*n* = 27) fearlessness based on mean (35.4) split for exploratory purposes. The shorter RTs of harming predicted stronger guilt ratings only in Low fearlessness (*r* =  − 0. 36, *P* = 0.03), but not in High fearlessness (*r* =  − 0. 07, *P* = 0.36) (Fig. 1C). In regards to the modulation effect of rebellious nonconformity on the association between RTs and guilt ratings, participants were divided into subgroups of relatively High (*n* = 30) and Low (*n* = 26) rebellious nonconformity based on mean (38.2) split. The shorter RTs during harming predicted also stronger guilt ratings in Low nonconformity (*r* =  − 0. 34, *P* = 0.04), but not in High nonconformity (*r* =  − 0. 28, *P* = 0.063). In order to examine the above-mentioned modulatory effects of psychopathy on the association between harming RTs and guilt ratings, we included and found that the fearlessness * harming RT (β =  − 0.207, *p* = 0.92) as well as rebellious nonconformity * harming RT (β =  − 0.062, *p* = 0.98) interactions did not yield significance.

Sensitivity tests were conducted to examine the effect of two outliers who reported a negative value of guilt ratings toward the action of harming others (Supplementary Table 1 and 2). The exclusion of outliers did not change the overall patterns of the results.

### Validity of the virtual obedience paradigm

The feeling of coercion was higher in the coercion group (6.49 ± 0.09; *p* < 0.001) than in the free-will control group (4.29 ± 0.22) for harming actions, but comparable for neutral actions (1.28 ± 0.2 vs. 1.0 ± 0.13; *p* = 0.26). In the free-will group, the average percentage of the participants who chose to be the agent of a harming action is 17 ± 3.0%, indicating that, in more than 80% of harming trials, participants chose to be just an observer instead of an agent. In the coercive group, participants were more reluctant to follow harming orders, showing longer RTs (3.13 ± 0.02, *p* < 0.001), as compared to neutral orders (3.01 ± 0.03).

### Neuroimaging results

Significant neuro-hemodynamic increase in the network of regions involved in the guilt experience and moral valence under the perpetrating immoral trials (*k* > 10, *P* < 0.05, FWE corrected). This network includes the anterior insula (AIC), temporal pole, dorsomedial prefrontal cortex (DLPFC), thalamus, postcentral gyrus, hippocampus, postcentral gyrus, superior frontal gyrus, and posterior cingulate. In addition, signal change was detected in the anterior midcingulate cortex (aMCC) (Table 3). Neural activations (harming vs. neutral) that significantly correlated with PPI-R total scores and guilt ratings during harming actions are listed in Table 4 and Supplementary Figure s1, including those from the aMCC, whose activations were related to experiences of guilt^46^.

**Table 3:** Neural Activations (harming vs. neutral) during the virtual obedience paradigm.

Regions	H	MNI coordinates			Peak T	Cluster size
		x	y	z		
**Harming > Neutral**						
Thalamus	L	− 12	− 24	8	5.32	2202
Anterior insula	R	30	10	− 16	4.84	617
Postcentral gyrus	R	26	− 40	74	4.64	230
Supramarginal gyrus	L	− 60	− 22	40	4.64	417
Anterior insula	L	− 34	6	− 14	3.51	785
Fusiform	L	− 42	− 54	− 12	4.49	96
Dorsomedial prefrontal cortex	R	2	62	16	4.39	365
Middle occipital gyrus	R	24	− 96	− 2	4.02	160
Postcentral gyrus	L	− 24	− 34	76	3.98	138
Hippocampus	R	20	− 24	− 12	3.95	38
Rolandic operculum	R	48	− 30	20	3.87	262
Middle occipital gyrus	L	− 52	− 68	− 10	3.86	131
Posterior cingulate	R	16	− 26	38	3.77	39
Superior frontal gyrus	R	18	− 10	76	3.76	38
Postcentral gyrus	R	38	− 38	62	3.69	79
Temporal pole	R	44	14	− 24	3.63	60
Anterior midcingulate cortex	R	8	6	34	3.1*	NA
**Harming < Neutral**						
NS						

Pooled group results (N = 56). All reported clusters significant at the FWE-corrected *P* < .05 level unless marked with an asterisk, which were taken from pre-defined regions of interest (ROIs) and significant at uncorrected *P* < .05.

H, hemisphere; MNI, Montreal Neurological Institute.

**Table 4:** Neural activations (harming vs. neutral) correlated with guilt and psychopathic traits from harming > neutral contrasts.

Regions	H	MNI coordinates			Peak T	Cluster size
		x	y	z		
**Guilt Ratings**						
Anterior midcingulate cortex	L	− 16	6	38	5.62	178
Anterior midcingulate cortex	R	14	4	34	4.78	98
Posterior cingulate/calcarine	R	24	− 68	12	5.26	878
Anterior insula	L	− 28	24	12	4.23	382
Anterior insula	R	36	18	14	3.82	36
Temporal pole	L	− 48	10	− 24	4.63	213
Inferior occipital gyrus	L	− 34	− 72	− 8	4.48	258
Anterior cingulate cortex	R	8	24	36	3.55	43
Dorsolateral prefrontal cortex	R	32	50	34	4.23	27
Insula	L	− 34	− 16	12	3.51	10
**PPI-R Total Scores**						
Anterior midcingulate cortex	L	− 16	6	34	− 4.51*	NA

Pooled group results (N = 56). All reported clusters significant at the FWE-corrected *P* < .05 level unless marked with an asterisk, which were taken from pre-defined regions of interest (ROIs) and significant at uncorrected *P* < .05. Negative and positive peak T-values represent negative and positive correlations, respectively.

H, hemisphere; MNI, Montreal Neurological Institute.

### Mediation results

#### Psychopathic traits mediate the relationship between experienced guilt and aMCC activity (harming vs. neutral)

**Figure 2:**
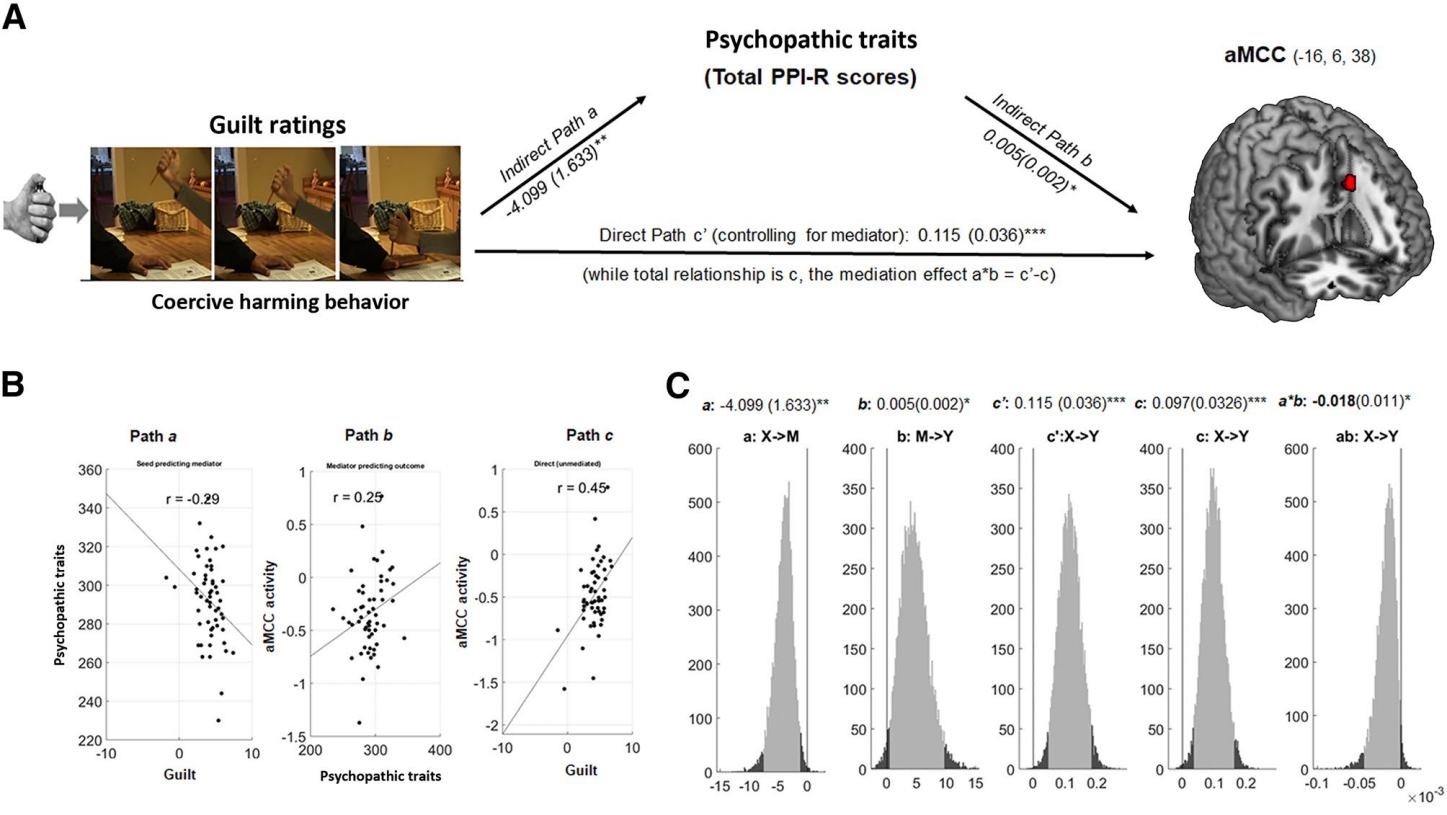
Psychopathic traits mediate the relationship between experienced guilt and aMCC activity (harming vs. neutral). (**A**) Path diagram demonstrates the relationship between variables in the path model. Guilt feelings (left) as the predictor variable predicts the hemodynamic activity in the aMCC (right). The connection of guilt ratings to the mediator (total PPI-R as psychopathic traits) as mediator is the a path. The lines are labeled with path coefficients, and standard errors are shown in parentheses. The connection of the mediator (total PPI-R) to the outcome (aMCC activity) is the b path. They are calculated controlling for guilt ratings, as the standard in mediation models. ∗  ∗  ∗ *p* < .001, ∗  ∗ *p* < .01, ∗ *p* < .05, two-tailed. The direct path is the c’ path, which is calculated controlling for brain mediator. (**B**) Substantiation of the mediation path a, b and c. Regression scatterplots depict the relationships between predictor (i.e., guilt) and psychopathic traits (path a). Partial regression scatterplots demonstrate the relationships between psychopathic traits and aMCC (path b). (**C**) The mediation effect (a ∗ b) is substantiated by the bootstrapped distributions. The range on the x-axis spanned by the lighter gray portion of the histogram is the 95% confidence interval for the effect.

While guilt feelings induced by coercive harming predicted higher activity in aMCC, psychopathic traits (total PPI-R) significantly mediate this guilt-brain association. During coercive harming, the psychopathic traits was negatively associated with guilt ratings and positively predicted aMCC activity: a =  − 4.1, Z =  − 2.91, b = 0.005, Z = 2.03 and a ∗ b =  − 0.02, Z =  − 2.01, all *P* < 0.05 (Fig. 2).

## Discussion

The present study addresses the link between neural correlates of guilt and psychopathic traits. We developed an fMRI virtual obedience paradigm, to simulate antisocial behavior under coercion, and to elicit first-hand guilt experiences in relation to psychopathic traits. Neural and behavioral results allow us to further elucidate the relationship between psychopathic traits and experienced guilt, and demonstrate how everyday psychopathic behavior in sub-clinical participants can be captured in the laboratory.

It is relieving to report that participants were overall taking longer RTs to initiate harming than to initiate neutral actions. They also reported significantly higher guilt responses to harming trials than neutral trials. However, it is interesting to note that participants who followed harming orders more promptly with shorter RTs later reported stronger feelings of guilt. This could be explained by their retrospective self-attribution of the lack of hesitation to callousness in delivering harm^47^, which in turn produces greater feelings of guilt.

The intercorrelations among conformity (RTs to a command), guilt feelings, and psychopathic traits found in this study using a general sub-clinical population might be less powered when predicting for the complex clinical problem of psychopathy. Here, participants who scored higher in rebellious nonconformity and fearlessness followed orders to both harming and neutral behaviors with longer and shorter RTs, respectively. It is likely for individuals with higher rebellious nonconformity to show a general non-compliance in following commands, regardless of the different types of actions. Those with higher fearlessness simply react faster, regardless of the varying types of orders. However, although High and Low psychopathic subgroups showed different patterns of harming RT-guilt associations based on median splits, the interactions of fearlessness * harming RT and rebellious nonconformity * harming RT did not yield significance. Whether this RT-guilt association differs in individuals with relatively higher and lower psychopathic traits among the general population remains an area for future inquiry.

Our findings remain consistent with the current literature. The neural activations (harming vs. neutral) that significantly correlated with PPI-R total scores and guilt ratings during harmful actions where those of the aMCC (see Table 4 and Supplementary Fig. 1), an area which is strongly associated with experiencing guilt^46^. Neural activations (harming vs. neutral) during the virtual obedience paradigm are also comparable to previously identified neural pathways associated with guilt^7,20–25,40^, while the present study further extends the literature of hypothetical and recollected guilt to include real-time guilt experiences. Likewise, subjective measures of guilt experience via the virtual obedience paradigm also successfully predicted psychopathic traits. Guilt ratings were negatively correlated with total PPI-R scores (*r* =  − 0.29), showing that less guilt during Harming trials was associated with higher psychopathic traits, which is in line with the long-established negative relationship between psychopathy and guilt.

Our findings implicate psychopathy as responsible for the inverse relationship between experienced guilt and guilt-related neural activation. More specifically, psychopathic traits were identified as a mediator of the association between guilt ratings and the aMCC activity. As previous findings have already established that the aMCC is a guilt-specific region^25,26,40^, and that its activity reflects the degree of aversive response people experience while perceiving others in pain and making harmful decisions during moral dilemma tasks^48,49^, we can infer that aMCC is important for deterring harmful actions.

Contrary to expectation, we did not find evidence that overall psychopathy measures predict antisocial behavior within subjects under coercion; instead, we identified an individual psychopathic trait that predicted greater willingness to harm. RTs during harming trials could not be used to differentiate accurately between participants who scored higher on the PPI-R and those who scored lower. However, grouping participants into High and Low fearlessness of psychopathic traits, we observed significantly shorter RTs during the harming in High fearless individuals (see Table 2). Notably, shorter RTs were previously associated with rewarding outcomes in individuals with psychopathy^50^, which suggests that, within High psychopathy, harmful actions are equally rewarding as neutral actions, whereas, within Low psychopathy, harmful actions are less rewarding than neutral actions. This is further supported by the observation that shorter RTs predicted stronger feelings of guilt for Low psychopathic but not for High psychopathic individuals. Low psychopathic individuals attribute more guilt to conceiving harmful actions as rewarding outcomes. Here, the present finding further points to subjective and neural measures for meaningful explorations to differentiate psychopathy.

A few limitations of the current work should be clarified for future research. For instance, the use of coercion in our experimental design is defined as the inability to account for voluntary harmful acts. While direct coercion by authority occurs outside of the laboratory setting, for example in the aforementioned war crimes, harmful acts happen via one’s own accord as well. However, situational factors also affect offenders’ decision to commit crimes^51^, which, from the perspective of the offender, can be a different albeit weaker type of coercion i.e. forced by circumstance or impulsions. Nevertheless, even under the assumption that the current study cannot address crimes committed willingly or as the result of other types of coercion, our study results provide an alternative explanation for why certain individuals are “enabled” by coercion when committing harmful actions. Finally, while participants taking the first-hand perspective to commit harming and neutral actions under the pressure of the experimenter showed heightened guilt feelings to harming, the fMRI effects found in the aMCC could be attributed to seeing different types of pictures, since activity in the aMCC also reflects the degree of the aversive response people experience while perceiving others in pain^37–39,44^. Additionally, while the aMCC has been deemed as a core region to be involved in executive functioning as well as pain perception, it was observed to exhibit disrupted activations in aggressive individuals in a recent meta-analysis study in which the consensus connectivity networks was delineated from meta-analytic connectivity modeling^52^. Participants with high and low executive functioning exhibited an opposite pattern of association between aMCC activation and aggression, suggesting that individual’s regulatory capability and aMCC functioning can modulate aggressive behavior that was induced by the psychological pain of social rejection^53^. While executive control might be less required in the present paradigm because of the coercive nature of the task, the aMCC activation could be reduced even more in individuals with high aggression as well as high psychopathic traits. Hence, an ideal design of a control condition in order to tease out guilt-effects from picture-effects –e.g., one where participants are presented with the harming scenarios but without them performing the order to continue– is warranted for future research.

All in all, this study shows that situational factors such as coercion and/or pressure from authority are able to encourage antisocial behaviors, as well as how the employment of tools such as the virtual obedience paradigm might be able to identify and elucidate certain elements that might predict everyday antisocial behavior. The next steps would be to test whether the lack of guilt as measured by the virtual obedience paradigm predicts future antisocial behaviors.

## Supplementary Information

Supplementary Information.

## Data Availability

The data that support the findings of this study and the code used for data analysis are available upon reasonable request to the corresponding author.
