# Supplementary material for: Psychopathic traits mediate guilt-related anterior midcingulate activity under authority pressure
Source: Sci Rep. 2021 Jul 21;11:14856. doi: 10.1038/s41598-021-94372-5 (PMC8295253; doi:10.1038/s41598-021-94372-5)
Supplement: Supplementary file 1 — Supplementary Information. [file 41598_2021_94372_MOESM1_ESM.docx]

**Psychopathic traits mediate guilt-related anterior midcingulate activity under authority pressure**

Yawei Cheng^1,2,3^†, Judith Chou^2^†, Roger Marcelo Martinez^4,5^, Yang-Teng Fan^6^, Chenyi Chen^4,7,8,9^*

1. Department of Physical Medicine & Rehabilitation, National Yang Ming Chiao Tung University Hospital, Yilan, Taiwan.

2. Institute of Neuroscience and Brain Research Center, National Yang Ming Chiao Tung University, Taipei, Taiwan.

3. Department of Education and Research, Taipei City Hospital, Taipei, Taiwan.

4. Graduate Institute of Injury Prevention and Control, College of Public Health, Taipei Medical University, Taipei, Taiwan.

5. School of Psychological Sciences, National Autonomous University of Honduras, Tegucigalpa, Honduras.

6. Department of Biological Science and Technology, National Chiao-Tung University, Taiwan.

7. Research Center of Brain and Consciousness, Shuang-Ho Hospital, Taipei Medical University, New Taipei City, Taiwan.

8. Graduate Institute of Mind, Brain and Consciousness, College of Humanities and Social Sciences, Taipei, Taiwan.

9. Psychiatric Research Center, Wan Fang Hospital, Taipei Medical University, Taipei, Taiwan

†Equally contributed to the study.

**Corresponding Authors**: Dr. Chenyi Chen

Graduate Institute of Injury Prevention and Control

Taipei Medical University

250 Wu-Hsing Street, Taipei 110, Taiwan

Tel: 886-2-2736-1661; Fax: 886-2-2739-0387

Email: [viniverson@gmail.com](mailto:viniverson@gmail.com); [chenyic@tmu.edu.tw](mailto:chenyic@tmu.edu.tw)

**Running title:** Experienced guilt in psychopathic traits.

**Number of words:** abstract (149 words, 4 key words), text (4795), 3 tables and 2 figures.

**Supplementary Materials**

**Figure s1: Neural correlates of experienced guilt under coercion (harming vs. neutral).** Neural response that correlated with guilt ratings when participants followed coercive commands to commit perpetrating trials. Guilt ratings during harming trials were positively correlated with activity in anterior midcingulate cortex (aMCC), anterior insular cortex (AIC), posterior cingulate/calcarine, temporal pole, dorsolateral prefrontal cortex (DLPFC), right temporoparietal junction (rTPJ), amygdala, and orbitofrontal cortex (OFC) (see Table 3, *P* < .01 for visual purpose).


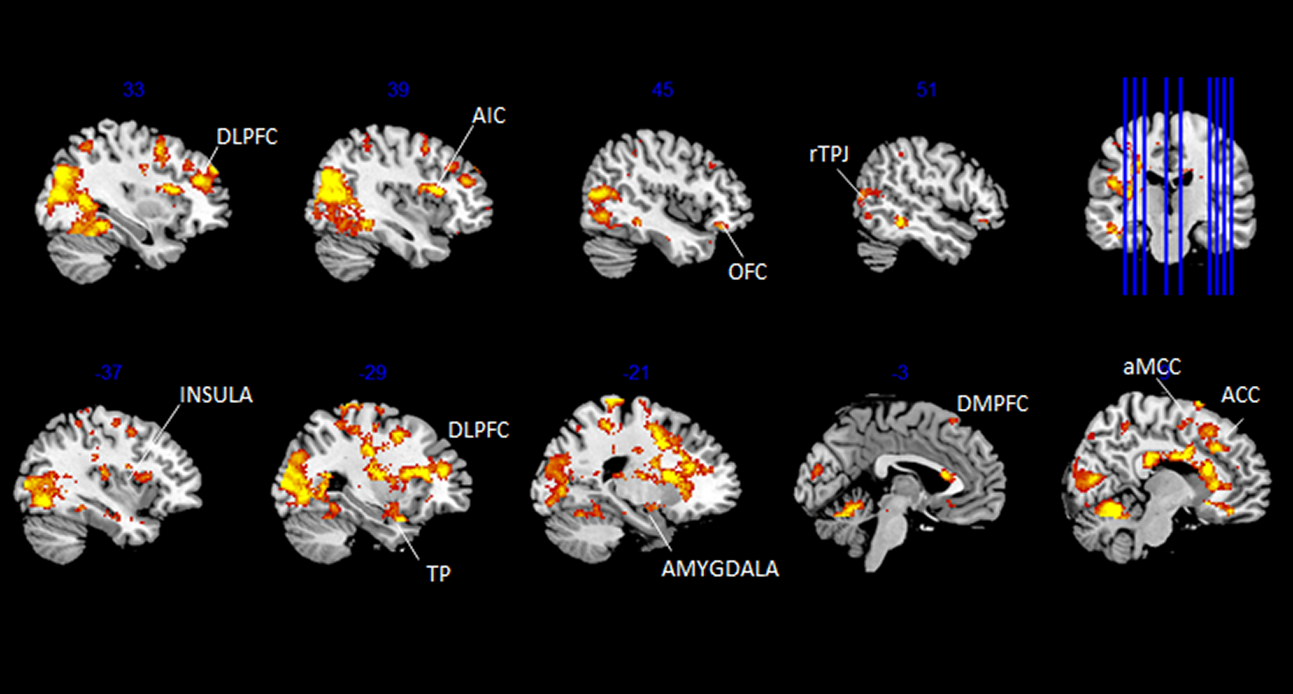


**Supplementary Table 1.** The psychopathy scores in two outliers (subject number#8 and #41) who reported a negative value of guilt ratings toward the action of harming others. C, cold heartedness; ME, machiavellian egocentricity; RN, rebellious noncomformity; BE, blame externalization; CN, carefree nonplanfulness; SOI, social influence; F, fearlessness; STI, stress immunity; PPI-R, psychopathic personality inventory-revised total score.

| Subjects | C | ME | RN | BE | CN | SOI | F | STI | PPI-R | percentile |
| --- | --- | --- | --- | --- | --- | --- | --- | --- | --- | --- |
| #8 | 32 | 44 | 51 | 36 | 39 | 33 | 41 | 23 | 299 | 64% |
| #41 | 33 | 44 | 39 | 31 | 43 | 49 | 36 | 49 | 304 | 75% |

**Supplementary Table 2.** Results of sensitivity tests on the correlations between RTs and guilt ratings before and after the exclusion of two outliers who reported a negative value of guilt ratings toward the action of harming others.

| Sample size | Harming RTs |  | Guilt Ratings |  |  |  |  |
| --- | --- | --- | --- | --- | --- | --- | --- |
|  | Mean ± SD |  | Mean ± SD |  | Pearson R | df | *P* value |
| Whole group N=56 (before removing 2 outliers) | 3.042 ± 0.204 |  | 4.132 ± 1.584 |  | -0.281 | 56 | .036 |
| Whole group N=54 (after removing 2 outliers) | 3.042 ± 0.204 |  | 4.33 ± 1.216 |  | -0.281 | 54 | .056 |
| High psychopathic fearlessness group N=27 (before removing 2 outliers) | 3.024 ± 0.156 |  | 3.941 ± 1.859 |  | -0.274 | 27 | .167 |
| High psychopathic fearlessness group N=25 (after removing 2 outliers) | 3.011 ± 0.155 |  | 4.352 ± 1.155 |  | -0.074 | 25 | .721 |
